# Anterior interosseous nerve syndrome following infection with COVID-19: a case report

**DOI:** 10.1186/s13256-023-03952-8

**Published:** 2023-06-11

**Authors:** Akira Ikumi, Yuichi Yoshii, Katsuya Nagashima, Yosuke Takeuchi, Masaki Tatsumura, Takeo Mammoto, Atsushi Hirano, Masashi Yamazaki

**Affiliations:** 1 Department of Orthopedic Surgery and Sports Medicine, Tsukuba University Hospital Mito Clinical Education and Training Center, Mito City, Ibaraki, Japan; 2 Department of Orthopedic Surgery, Institute of Medicine, University of Tsukuba, Tsukuba city, Ibaraki, Japan; 3 Department of Orthopedic Surgery, Tokyo Medical University Ibaraki Medical Center, 3-20-1 Amimachi, Inashiki-Gun, Ibaraki 300-0395 Japan

**Keywords:** Coronavirus disease 2019, Anterior interosseous nerve syndrome, Tendon transfer

## Abstract

**Background:**

Various neurological manifestations associated with coronavirus disease 2019 have been increasingly reported. Herein, we report a rare case of anterior interosseous nerve syndrome, which occurred 5 days after the onset of coronavirus disease 2019.

**Case presentation:**

A 62-year-old Asian woman with a history of coronavirus disease 2019 who developed a complete motor deficit in the left flexor pollicis longus and pronator quadratus without sensory deficits. The symptoms appeared as a sudden onset fatigue and severe pain of the left arm, 5 days after the onset of coronavirus disease 2019. She noticed paralysis of the left thumb at 2 weeks after the onset of coronavirus disease 2019. Electromyography assessment of the anterior interosseous nerve-dominated muscles revealed neurogenic changes such as positive sharp wave and fibrillation in flexor pollicis longus and pronator quadratus, confirming the diagnosis of anterior interosseous nerve syndrome. There were no other diseases that could have resulted in peripheral nerve palsy. We performed a functional reconstruction surgery of the thumb by tendon transfer from the extensor carpi radialis longus to the flexor pollicis longus. The patient reported a good patient-reported outcome (2.27 points in QuickDASH Disability/Symptom scoring and 5 points in Hand20 scoring) at final follow-up (1 year after the surgery).

**Conclusion:**

This case highlights the need for vigilance regarding the possible development of anterior interosseous nerve syndrome in patients with coronavirus disease 2019. Tendon transfer from extensor carpi radialis longus to flexor pollicis longus can provide good functional recovery for unrecovered motor paralysis after anterior interosseous nerve syndrome.

## Background

Since the coronavirus disease 2019 (COVID-19) pandemic started in 2020, it has become increasingly evident that several neurological manifestations are associated with COVID-19, involving both the central and peripheral nervous systems [1].

Anterior interosseous nerve syndrome (AINS) represents a form of neuralgic amyotrophy (Parsonage–Turner syndrome) [2]. It typically presents with a prodrome of arm and/or forearm pain lasting hours or days, followed by a flexion palsy of the thumb [flexor pollicis longus (FPL)], with or without distal interphalangeal joint flexion of the index finger [flexor digitorum profundus index (FDP-I)] and/or forearm pronation [pronator quadratus (PQ)] [3]. The two most common causes of this syndrome are compressive neuropathy within the forearm and idiopathic immune-mediated peripheral neuritis [4]. AINS is commonly preceded by an antecedent stressful event or trigger, such as surgery, trauma, strenuous exercise, vaccination, pregnancy, or viral illness [5].

Although cases of neuralgic amyotrophy following COVID-19 infection have been reported [6–8], AINS, which causes paralysis only in muscles more distal than general neuralgic amyotrophy, has not been described in a patient with COVID-19. Herein, we report a rare case of AINS suspected to be triggered by COVID-19 infection.

## Case presentation

A 62-year-old right-handed Asian female swimming coach reported to our hospital with the complaint of difficulty in flexing her left thumb. She had a history of sudden onset of fever, cough, and diarrhea developed in August 2021. She had no relevant past medical history. She tested positive for SARS-CoV-2 using a nasopharyngeal swab reverse transcription–polymerase chain reaction (RT–PCR). She received symptomatic treatment at home. She recovered from respiratory and gastric symptoms within 10 days after the onset of COVID-19.

Five days after the onset of COVID-19, she had sudden onset fatigue and severe pain on the left arm. Although the fatigue and pain decreased spontaneously approximately after 1 week, she noticed paralysis of the left thumb at 2 weeks after the onset of COVID-19. Due to persistent difficulty in flexing the left thumb, she came to our hospital at 2 months after the onset of paralysis.

**Fig. 1:**
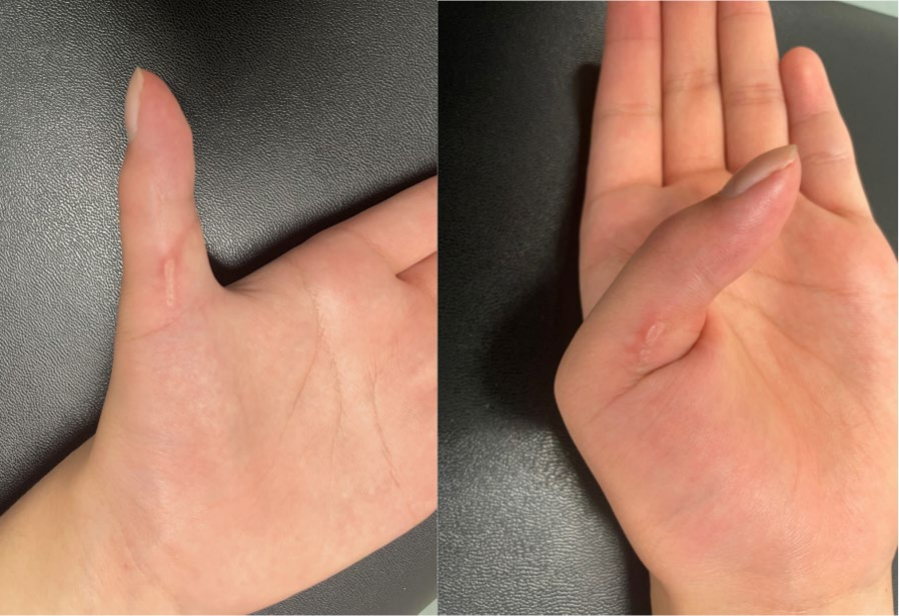
Active thumb movement at 6 months after the onset of symptoms. No thumb interphalangeal joint flexion was observed (MMT grade 0). *MMT* manual muscle test

On physical examination, neither active flexion of the thumb interphalangeal joint nor joint contracture (passive range of motion was 30° in extension and 80° in flexion) was observed. **(**Fig. 1**)** There was no sensory disturbance and Tinel’s sign at her left upper extremity. Tenderness at the ulno–volar area of elbow was observed. Manual muscle test (MMT) result of the anterior interosseous nerve (AIN)-dominated muscle showed grade 0/5 in FPL and PQ, and 5/5 in flexor digitorum profundus for the index and middle digits (FDP-I and FDP-M).

No abnormal findings were found on plain radiograph or computed tomography. Magnetic resonance imaging (MRI) revealed no intensity change indicating inflammation around the median nerve and AIN area. No occupying lesion such as tumor or cyst compressing nerve was observed around the median nerve and AIN. No characteristic sign of AINS such as bullseye sign in MRI or torsion in ultrasonography, which indicate nerve or fascicle constriction, were observed. No abnormal findings suggesting autoimmune disease were observed from blood testing.

**Fig. 2:**
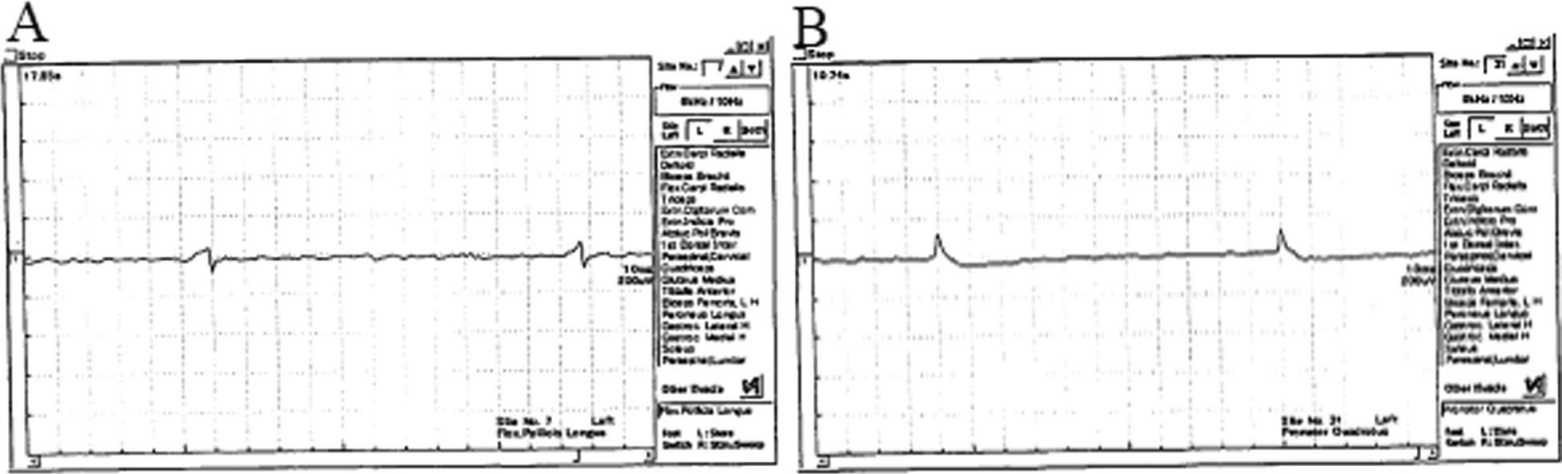
Electromyography at 3 months after the onset of symptoms. Left FPL (**A**) and PQ (**B**) demonstrate denervation (positive sharp wave) without active discharge. *FPL* flexor pollicis longus, *PQ* pronator quadratus

Electrophysiological studies (nerve conduction test and electromyography) were performed at 3 months after the onset of symptoms. There were no abnormal findings in the median nerve conduction test. Electromyography of the AIN-dominated muscles revealed neurogenic changes such as positive sharp wave and fibrillation in the FPL and PQ (Fig. 2).

From the above findings, we diagnosed the case as AINS. In the absence of recovery in the left thumb paralysis at 6 months after symptom onset, we proceeded with functional reconstruction surgery of the thumb by tendon transfer from the extensor carpi radialis longus (ECRL) to the FPL.

**Fig. 3:**
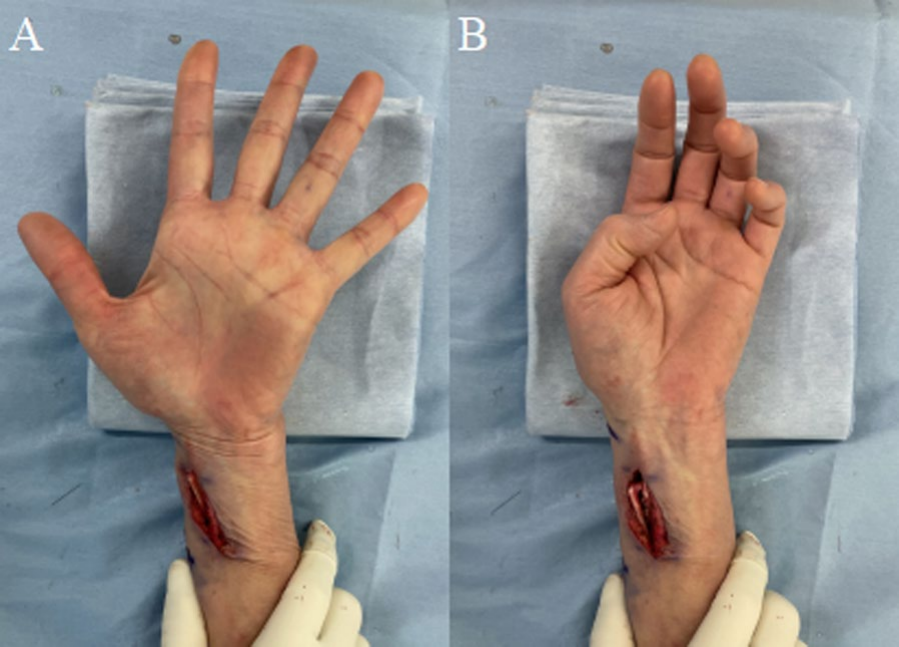
Intraoperative findings. Smooth active thumb extension (**A**) and flexion (**B**) is observed after performing tendon transfer from ECRL to FPL. *ECRL* extensor carpi radialis longus, *FPL* flexor pollicis longus

The surgery was performed under wide-awake local anesthesia no tourniquet (WALANT) [9]. The ECRL tendon was cut at the attachment of second metacarpal bone; the distal end of ECRL tendon was then passed through the subcutaneous tunnel from dorsal to volar side of the forearm, and finally it was transferred to FPL tendon by interlacing suture technique performed three times at 3 cm proximal to the wrist. To decide the tension of each tendon, a single interlacing suture was performed under maximum passive traction in the wrist neutral position. We instructed the patient to move her thumb interphalangeal joint. The final tension was decided based on whether the patient was able to perform tip pinch movement completely and extend thumb metacarpophalangeal and interphalangeal joint completely in wrist neutral position. After deciding the suture tension, additional interlacing suture was performed (sutured three times in total). Smooth active flexion of the thumb was easily confirmed intraoperatively using the WALANT technique (Fig. 3).

**Fig. 4:**
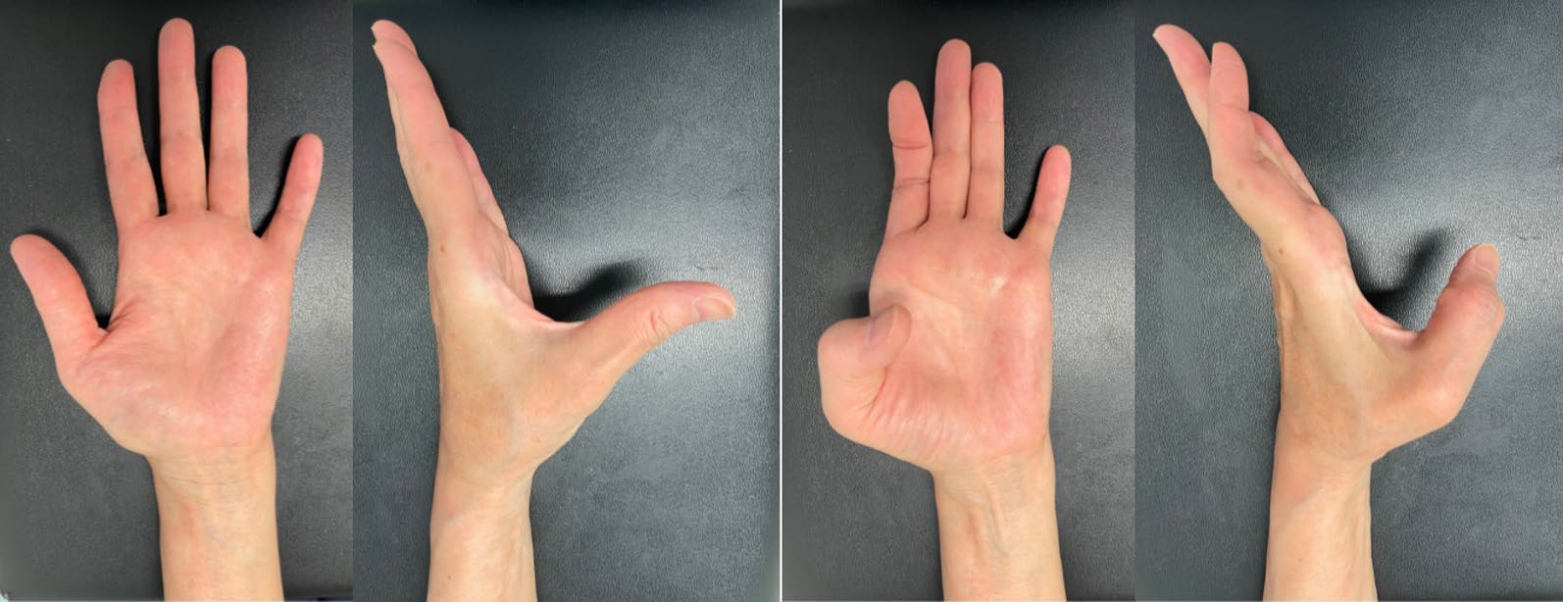
Thumb active motion at final follow-up

A thumb spica splint of the wrist in neutral position and thumb in volar-abduction position was applied for 3 weeks after surgery. Active range of motion exercise of the thumb interphalangeal joint was started immediately after surgery with a hand therapist. At final follow-up (1 year after surgery), the active range of motion of the thumb was 0–60° in metacarpophalangeal joint and −6° to 66° in interphalangeal joint. Pinch strength improved over 80% on the ipsilateral side; R3.5/L3.0 kg in tip pinch and R6.2/L5.2 kg in key pinch. Patient-reported outcomes were good (2.27 points in QuickDASH Disability/Symptom scoring and 5 points in Hand20 scoring) (Fig. 4).

## Discussion and conclusions

In this case, the prodromal symptoms (fatigue and severe pain in the upper arm) developed after 5 days from the onset of COVID-19, and symptoms of AINS developed after the prodromal symptoms subsided. There was no history of autoimmune disease, and no occupying lesion compressing nerve was observed from the results of various imaging studies. In the absence of any other onset possible cause for the observed peripheral nerve palsy, the COVID-19 infection was suspected to have triggered the onset of AINS.

Although the main symptoms of COVID-19 infection are fever, respiratory disease (upper respiratory tract inflammation or pneumonia), and gastrointestinal disease (diarrhea), COVID-19 also causes neurological symptoms in more than one-third of patients [10]. Although the COVID-19 neurologic involvement has been described mainly with the central nervous system manifestations such as anosmia, ageusia, and headache, Guillain–Barre syndrome (GBS), neuralgic amyotrophy (NA), and polyneuropathy of the peripheral nervous system has also been described [11]. Involvement of peripheral nervous system in patients with COVID-19 may result from direct neuro-invasion or from an autoimmune postinfectious mechanism [10]. Although there are no reports examining the time from COVID-19 infection to the onset of symptoms in NA, Aladawi *et al*., in their systematic review, described an average time of approximately 2 weeks for the onset of GBS after COVID-19 infection [12]. Furthermore, the time from COVID-19 infection to onset of GBS symptoms was reported to be less than 1 week only in few cases; therefore, they have described that a postinfectious autoimmune reaction was the main mechanism of GBS development in COVID-19 infection. Although the period between the onset of COVID-19 symptoms and prodromal symptoms of AINS in this case was 5 days (less than 1 week), COVID-19 often has an asymptomatic period in its early stage. Postinfectious autoimmune mechanisms were mainly involved in the development of AINS in this case, considering the possibility that the actual infection developed several days before the onset of symptoms.

With regards to AINS treatment, nerve decompression within the forearm has not been shown to improve outcomes [5, 13]. On the other hand, several studies reported good functional recovery of patients with AINS after performing intrafascicular neurolysis in the upper arm [3, 14]. However, the usefulness of early surgical intervention is still controversial because two-thirds of patients with AINS recovered spontaneously within 3 months of onset without any treatment [3, 13, 15]. Yamamoto *et al*. reported that internal neurolysis was found to be superior to nonoperative treatment in patients with AINS who showed no recovery after 3 months [16]. Feinberg *et al*. also demonstrated that axonal regeneration should consistently begin by at least 6 months after the onset of symptoms [17]. Functional reconstruction surgery (tendon transfer from ECRL to FPL) was performed in this case as there was little improvement in paralysis at 6 months after the onset of symptoms. Fascicular constrictions (FCs) or hourglass constrictions (HGCs) of the median nerve are often marked by focal narrowing of a nerve or a nerve fascicle in patients with AINS [18, 19]. Although several reports have described MRI and ultrasonography as useful tools to detect FCs or HGCs in patients with AINS [18, 20], these constriction findings could not be detected in this case. After discussing with the patient, we chose functional reconstruction surgery without internal neurolysis. As a result, the patient recovered well from thumb dysfunction. Although tendon transfer with preservation of the FPL tendon allowed for the possibility of spontaneous recovery of AINS, the FPL and PQ was still completely paralyzed until final follow-up. If a patient with AINS desired early certain functional recovery and no recovery sign showed even after 6 months of the onset, functional reconstruction surgery such as tendon transfer without internal neurolysis should be considered as one of the treatment choices.

In summary, our case highlights the need for vigilance regarding the possible development of AINS in patients with COVID-19. Physicians should be aware that peripheral neuropathies such as AINS may occur after COVID-19, which can help to avoid delay in diagnosis and allow for early management. Tendon transfer from the ECRL to the FPL can provide good functional recovery for unrecovered motor paralysis after AINS.

## Data Availability

Not applicable.

## Abbreviations

AIN: Anterior interosseous nerve
AINS: Anterior interosseous nerve syndrome
COVID-19: Coronavirus disease 2019
ECTR: Extensor carpi radialis longus
FC: Fascicular constrictions
FDP-I: Flexor digitorum profundus index
FPL: Flexor pollicis longus
GBS: Guillain–Barre syndrome
HGC: Hourglass constrictions
MMT: Manual muscle test
MRI: Magnetic resonance imaging
NA: Neuralgic amyotrophy
PQ: Pronator quadratus
WALANT: Wide-awake local anesthesia no tourniquet
